# dictyBase 2026: a reimplementation of the *Dictyostelium* model organism database on minimal infrastructure

**DOI:** 10.21203/rs.3.rs-10986412/v1

**Published:** 2026-09-10

**Authors:** Maddox Mendieta, Robert J. Dodson, Siddhartha Basu, Rex L. Chisholm, K. Matthew Scaglione

**Affiliations:** 1 Department of Molecular Genetics and Microbiology, Duke University School of Medicine, Durham, NC, USA; 2 Center for Genetic Medicine, Feinberg School of Medicine, Northwestern University, Chicago, IL, USA.; 3 Department of Cell Biology, Duke University School of Medicine, Durham, NC, USA

**Keywords:** Dictyostelium discoideum, model organism database, biocuration, community curation, comparative genomics, research data infrastructure

## Abstract

*Dictyostelium discoideum* is a model organism used to investigate a wide array of biological processes including chemotaxis, cell motility, phagocytosis, multicellular development, and autophagy. Many of its genes have human orthologs implicated in disease, making it a useful model organism for studying conditions ranging from cancer to neurodegeneration. dictyBase has served this community for two decades as a home for genomic data, protocols, a stock center, and community news. Here we describe a complete reimplementation of dictyBase as a self-contained service that uses static data files rather than a database server. Each gene record gathers curated function, human-disease context, developmental expression, and protein structure in a single webpage. The site also provides tools for sequence search, genome browsing, enrichment analysis, and experimental design. The complete Dicty Stock Center (DSC) catalog of 7,055 strains and 1,265 plasmids is included, and both can be ordered from the site. New literature is curated by an author-in-the-loop pipeline. A language model drafts the annotations, the authors of the paper correct them, and a curator approves them. Analyses can be run on curator-approved annotations alone or extended to include those still awaiting review. dictyBase is freely available at https://dicty.org.

## Graphical Abstract

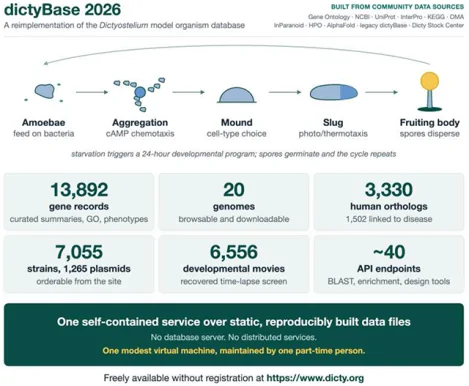

## Introduction

The social amoeba *Dictyostelium discoideum* is a widely used model in cell and developmental biology. In its fed state *Dictyostelium* is a unicellular amoeba that consumes bacteria, however, under conditions of nutrient deprivation *Dictyostelium* undergoes a developmental process that results in the formation of a multicellular fruiting body within approximately 24 hours (Figure 1). This developmental process makes *Dictyostelium* a uniquely tractable system for the study of chemotaxis, cell motility, cell–cell signaling, cell-type differentiation, and morphogenesis [1]. In addition, a substantial fraction of *Dictyostelium* genes have human orthologs allowing *Dictyostelium* to serve as a model for studying a wide range of human diseases. This includes using *Dictyostelium* to investigate cellular processes disrupted in conditions ranging from the neuronal ceroid lipofuscinoses and disorders of autophagy, to host-pathogen interactions and neurodegenerative disease [2-8]. These studies are well suited for *Dictyostelium*, which has a number of experimental advantages. These include a compact haploid genome, efficient homologous recombination and CRISPR-based genome editing, and inexpensive culturing. These and other features make *Dictyostelium* a valuable model organism for biomedical research.

For two decades, dictyBase has served as the central model organism database (MOD) for *Dictyostelium* and related Dictyostelids [9-13]. It provides the community's approved gene nomenclature, expert literature curation, Gene Ontology, strain and phenotype annotations, genome sequences and assemblies, and through the Dicty Stock Center, a repository from which strains and plasmids are distributed worldwide. These resources underpin a large fraction of published *Dictyostelium* research and are relied upon for gene naming, functional annotation, and reagent access.

Like other model organism databases, dictyBase must sustain a comprehensive, continuously updated resource despite changes in community size and uncertain funding [14]. Many modern MODs use distributed, service-oriented architectures that require specialized expertise and continuing engineering support. This effort is difficult to sustain for smaller communities that lack multi-million-dollar budgets.

We rebuilt dictyBase from scratch to expand its functionality while reducing operating cost and technical complexity. dictyBase now serves gene records, comparative and human-disease data, transcriptomic and proteomic expression, and its analysis tools from a single self-contained service backed by static data files. A documented set of scripts rebuilds those files from community sources including the legacy dictyBase, the Gene Ontology Consortium, NCBI, UniProt, InterPro, and KEGG. The running system needs no database server and no distributed services. The new site adds human-ortholog and disease integration, faceted gene discovery, additional analysis and molecular-biology design tools, and an education module for undergraduate and high school teaching. This implementation provides a practical, low-cost approach for maintaining a full-featured MOD with a small team.

## Data content and sources

### Gene records and nomenclature.

dictyBase provides records for the 13,892 protein-coding genes of the *D. discoideum* AX4 reference genome, keyed by stable DDB_G identifiers and the community's approved gene symbols. Each record integrates a curated summary where available, gene models derived from the reference annotation, cross-references to NCBI, UniProt, and related resources, and downloadable genomic, coding, and protein sequences. dictyBase remains the authority for *Dictyostelium* gene nomenclature.

### Function: Gene Ontology and phenotypes.

Gene Ontology annotations are imported from the Gene Ontology Consortium's *D. discoideum* release [15] and displayed grouped by aspect (molecular function, biological process, cellular component) with supporting evidence codes and references. In total, 8,659 of the 13,892 genes carry at least one Gene Ontology annotation. Each annotation carries a provenance badge distinguishing expert dictyBase curation from electronic inference (UniProt, InterPro, GO_Central), so users can weight annotations by evidence quality. Curated loss-of-function phenotypes are provided for 1,197 genes, each with associated experimental conditions and literature.

### Comparative genomics and human disease.

To facilitate studies of conserved disease mechanisms, dictyBase links *D. discoideum* genes to their human orthologs and, where applicable, to human disease. Orthology is derived from the OMA browser [16] and InParanoid [17], and disease associations from OMIM and Orphanet via the Human Phenotype Ontology [18]; 3,330 genes have an assigned human ortholog and 1,502 are linked to a human disease (for example, the *cln5* ortholog of human *CLN5*, mutated in neuronal ceroid lipofuscinosis). A *Dictyostelium* gene can map to more than one human protein, so the two counts differ. The 3,330 genes carry 5,176 gene-ortholog pairs. Of these, 1,554 are one-to-one, 831 one-to-many, 221 many-to-one and 1,577 many-to-many. The remaining 993 come from InParanoid alone, which does not assign a relationship type. Each record reports the relationship type together with the supporting method. Both methods call 1,869 pairs, OMA alone calls 2,314 and InParanoid alone 993. In all, 1,830 genes have at least one ortholog corroborated by both methods.

A disease association belongs to the human gene. It reaches the *Dictyostelium* gene through that mapping, not through any *Dictyostelium* evidence. The 1,502 disease-linked genes therefore correspond to 1,994 gene-ortholog pairs and 5,282 disease-term annotations. Only 638 of those genes are linked through a one-to-one ortholog. Where a gene maps to several human paralogs, the inherited terms may be unrelated to one another. abcA2 maps to ABCA1, ABCA3, ABCA5 and ABCA7. It therefore inherits terms as different as Tangier disease, pulmonary surfactant metabolism dysfunction and susceptibility to Alzheimer disease. These associations are leads for hypothesis generation rather than validated *Dictyostelium* disease models. Within-species paralog and gene-family relationships are computed on demand by sequence comparison.

### Transcriptomics and proteomics.

Developmental gene-expression profiles across the aggregation-to-fruiting time course are provided from RNA-seq [19-21] and are used both to compute co-expression relationships and to overlay expression tracks in the genome browser. Two mass-spectrometry proteomic datasets are integrated: a developmental proteome spanning five life-cycle stages (4,502 proteins) [22] and an insoluble development and heat-stress proteomes (8,043 proteins) [23].

### Pathways.

Metabolic and signaling pathway memberships are imported from KEGG [24], covering 1,927 genes across 131 pathways, and are surfaced both on each gene record and as a term set for enrichment analysis.

### Interactions.

Curated physical and genetic interactions are imported from BioGRID [25], covering 85 interactions among 66 genes. Each protein record additionally displays the predicted association network for that gene from STRING [26].

### Protein structure and domains.

Protein records display InterPro domain architectures [27] as annotated diagrams, embed AlphaFold predicted structures [28, 29] where available, and present curated post-translational modification annotations.

### Genomes.

dictyBase hosts the assemblies and annotations of 20 sequenced dictyostelid genomes, spanning the taxonomic groups of the social amoebae. Among these genomes, the genomes of three wild isolates of *Dictyostelium* are included [30]. All assemblies can be viewed in the integrated genome browser or downloaded as FASTA, with gene models for 16. Nineteen are searchable by blast. By pairing reference genomes with a growing collection of *D. discoideum* and related wild isolates, dictyBase enables population-genomic and strain-level comparisons that a single reference genome cannot support.

### The Dicty Stock Center.

The complete Dicty Stock Center catalog is integrated directly into the resource. This includes details of 7,055 strains of which 2,130 are available, and ~21,500 genome-wide (GWDI) knockout strains are available and accessible via the search function. In addition, 1,265 plasmids are available along with 19 bacterial strains. Users assemble orders in a cart and submit them through an integrated request workflow.

### A high-throughput developmental phenotype screen.

dictyBase incorporates the time-lapse screen of Sawai and colleagues [31]. In that work 2,257 insertional mutants were filmed through development and scored across stages including during normal growth, cAMP wave propagation, aggregation, mound, slug, and culmination. 387 strains show a defect in at least one stage. dictyBase now hosts 3,317 imaging runs as 6,556 playable movies. Where the disrupted gene is known the record links to it, covering 337 strains across 382 genes. Of the genes screened there are 1,111 strains across 269 genes that can be ordered for analysis.

### A separated machine-generated annotation layer.

Automated annotation increases coverage but must remain distinguishable from curated evidence. Accordingly, dictyBase keeps a machine-generated annotation layer, covering 1,374 genes, separate from curated and community-contributed annotations. These annotations are visually badged, individually toggleable, and kept distinct from expert-curated evidence. Every gene symbol and GO identifier in the layer is validated against the reference gene and Gene Ontology sets during generation.

## Implementation and architecture

### Design overview.

dictyBase is implemented as a single self-contained web service written against the Python standard library, serving a vanilla-JavaScript single-page application (Figure 2). The system uses no external database server, no message broker, and no distributed services. The data are structured, versioned, rebuilt from source, and queryable through the API, but they are not managed by a database management system. It also has no build step, and its only runtime dependency for core function is a Python interpreter.

### Data as versioned static builds.

dictyBase does not serve data from a live database. It serves pre-computed, versioned data files in JSON and standard bioinformatics formats. A documented suite of build scripts regenerates these from primary community sources including the Gene Ontology Consortium, NCBI, UniProt, InterPro, KEGG, the OMA browser, InParanoid, the Human Phenotype Ontology, AlphaFold, and the dictyBase collection. The resulting datasets can therefore be reproduced from their sources. The running service reads these assets directly, compressing text responses on the fly. To update a dataset, a curator reruns the relevant build script and redeploys the resulting files; the application itself does not need to change.

### Access and computation.

In addition to the web interface, dictyBase exposes a public REST API of approximately 40 endpoints providing programmatic access to gene records, search, sequence retrieval, and each analysis tool. Every analysis runs inside the same process. Sequence retrieval reads directly from the genome assembly and gene models. Over-representation analysis is a self-contained hypergeometric test, with Benjamini–Hochberg control of the false-discovery rate. The molecular-biology utilities utilize the genomic data in the same way. Designing a guide RNA screens the whole assembly for off-targets, and primer design and codon optimization draw on the same sequence data. Access does not require an account, and the site stores no personal information about readers. Curators hold named accounts, and submitting authors provide contact details. Usage is measured with a cookieless, first-party analytics counter that records only aggregate, bucketed page categories.

### Operational footprint.

The service and its static assets run on a single modest virtual machine. Most content could also be served as static files behind a small dynamic backend. The complete source code and data-build scripts are openly available (see Availability), enabling independent deployment, inspection, and reuse.

### AI-assisted software development.

The software described here was written with substantial assistance from an AI coding assistant (Anthropic's Claude, used through the Claude Code command-line interface [Opus 4.8]). The assistant was used to draft and refactor the service, the single-page application, and the data-build scripts, under continuous direction and review by the authors. With this assistance, the build time for the website is drastically reduced.

The authors reviewed, tested, and revised each component before use. An automated test suite checks the software, and the build process validates served data against their primary sources. For example, every gene symbol and Gene Ontology identifier is checked against the reference sets to ensure that identifiers are valid. Because the full source is openly available (see Availability), the implementation can be audited independently. The data were also validated manually by spot checking data for 30 random genes.

The use of a language model for software development is distinct from the machine-generated annotation layer described above. That layer is separately labeled, can be hidden, and is not presented as curated evidence. Gene nomenclature and the expert Gene Ontology and phenotype annotations that make up the curated record are human authored. A language model does draft candidate annotations from newly published papers, as described under Curation and community, but these annotations are held until a curator approves them.

## Web interface and analysis tools

### The gene record.

Each gene has a single record, organized into tabbed sections: summary, Gene Ontology, phenotypes, literature, protein structure, interactions, orthologs, and post-translational modifications. Each section carries cross-references to the external resources it draws on. Gene symbols throughout the site are linked and augmented with hovercards that display a gene's name, summary, and key attributes without navigation, and any gene can be cited or added to a personal workspace (below). The literature section additionally lists the investigators who have published on each gene, identified from the senior authors of its papers and refreshed automatically as new work is indexed, with a curator-reviewed mechanism for research groups to add themselves (Figure 3).

### Search and discovery.

dictyBase offers general full-text search across genes and site content, together with focused searches by curated phenotype, Gene Ontology term, and subcellular localization. An advanced gene finder supports filtering of the entire catalog by combinations of properties including curated phenotype, the presence of a human ortholog, disease association, and developmental expression peak and allows for the export of the resulting gene set. A search function provides rapid navigation to any gene, page, or tool.

### Analysis tools.

dictyBase provides several tools for analyzing genes and sequences. Sequence-similarity search is available through BLAST [32], spanning nucleotide and translated searches of the genome assemblies as well as protein searches against the reference proteome, with hits linked to their gene record; a genome browser built on IGV.js [33] displays the 20 dictyostelid genome assemblies with gene models and developmental RNA-seq tracks [19-21]. Gene-set analyses include Gene Ontology, phenotype, and KEGG pathway over-representation testing; multi-gene developmental expression comparison; and identification of co-expressed genes. For experimental design, dictyBase provides guide-RNA design with genome-wide off-target screening, quantitative-PCR primer design with parameters tuned for the organism's AT-rich genome, and codon optimization against *Dictyostelium* codon usage. On the protein record, domain architectures, cross-species conservation, and AlphaFold predicted structures are displayed interactively. The developmental phenotype screen has its own browser, which presents the six-stage profiles as a clustered heatmap, plays the movies inline, and exports the filtered set as a table. A gene identifier converter maps between gene symbols, DDB_G accessions, UniProt accessions and NCBI Gene identifiers. Sequence utilities retrieve genomic regions, perform in-silico PCR, and build multiple sequence alignments across the hosted genomes. A batch annotator returns a downloadable table of selected annotation fields for a list of genes, and a gene-set report combines enrichment, human-ortholog and disease overlap, and developmental expression profile into a single summary. dictyBase also distributes community-contributed analysis software, currently a Fiji plugin that tracks cell centroids through a time-lapse series and an accompanying spreadsheet template for chemotaxis analysis [34].

### From a human disease gene to a reagent.

Consider a researcher who studies the neuronal ceroid lipofuscinoses and wants to know whether *Dictyostelium* is worth adopting. Searching *CLN5* reaches the *cln5* record, where the orthologs section reports a one-to-one assignment supported by both OMA and InParanoid and links the gene through the Human Phenotype Ontology to the adult, juvenile, and late-infantile forms of CLN5 disease. The developmental RNA-seq profile shows *cln5* expressed during growth and through early development and declining thereafter, and the phenotypes section lists four curated loss-of-function phenotypes for *cln5*-null strains, among them decreased cell-substrate adhesion and decreased chemotaxis to cAMP. The page also provides reagents related to the gene of interest. In the case of *cln5* this includes a published, curated *cln5*-null strain not housed in the DSC and three GWDI insertion mutants of *cln5* that are in stock. Available strains can be added directly to the ordering cart. A laboratory seeking a defined null in its own strain background can run the guide-RNA designer on the same page to generate SpCas9 guides scored for off-targets across the whole genome.

### From a sequence to a conservation profile.

Users can also begin with a sequence rather than a gene name. A BLAST search against the hosted assemblies returns hits annotated with the *D. discoideum* gene they fall within. This search also opens the locus in the genome browser alongside neighboring genes and the developmental RNA-seq track. From that record, the protein can be compared across species or among wild isolates. A translated search across the sequenced dictyostelids returns the best hit in each genome. Each hit displays its percent identity and alignment length. A wild-isolate comparison reports the orthologous locus in each sequenced isolate, its percent identity, and the individual amino-acid substitutions relative to AX4. For example, across the sequenced isolates cln5 differs from AX4 at only a few residues. This pattern is consistent with a conserved housekeeping gene. tgrC1, one of the self/non-self recognition genes, however, differs at more than two hundred residues in one isolate. In another, the closest locus is the paralog tgrC5 rather than an allele. Together, these comparisons distinguish conserved from hypervariable genes and allelic variation from paralogy. They are available from every gene record.

### A personal workspace.

Users may collect genes of interest into a per-browser workspace ("basket") that persists locally without an account. The collected set can be submitted directly to the enrichment and expression-comparison tools or exported as tabular data or protein FASTA.

### Education.

dictyBase also includes an education module for undergraduate and high school teaching. It contains an interactive depiction of the life cycle linked to marker genes, a glossary, a self-assessment quiz, a set of original, openly licensed teaching figures, and a library of 30 classroom laboratory protocols. This includes a full semester college course on the cell biology of *Dictyostelium discoideum*.

## Curation and community

### Curation model.

dictyBase distinguishes five annotation layers including expert curation performed by dictyBase, author curation of newly published papers, community-contributed annotations, a set of manually produced computational predictions, and the separated machine-generated layer described above. The four non-expert layers are shown as unreviewed or provisional until a curator approves them. Each layer has a separate badge and display control, allowing users to identify the source and evidence basis of every annotation.

### Curation interface.

A web-based curator interface allows authorized curators to add and edit gene and stock-center records directly. In addition to free-text summaries, curators enter Gene Ontology terms with evidence codes and references, mutant phenotypes with experimental conditions and references, and approved gene nomenclature. These entries appear on the record as expert-curated annotations. A literature-driven to-do view surfaces genes that have published work but no curated summary, ranked by citation count, to focus curation effort. Edits are stored as durable overrides that are preserved across data rebuilds, so that manual curation is never overwritten when underlying assets are refreshed from source. Community members may submit gene annotations, corrections, and datasets through structured forms for curator review. The site also preserves community materials from the established resource, including a searchable archive of the Dicty electronic mailing list spanning questions and answers contributed since 1997. dictyBase also maintains the shared community infrastructure. This includes a searchable directory of *Dictyostelium* laboratories, the archive of the annual international *Dictyostelium* conferences, a listing of open positions in the field, and the record of community award recipients.

### Author-in-the-loop literature curation.

Literature curation requires substantial curator time. dictyBase therefore uses a language model to generate draft annotations that the authors of the paper review and correct. New *Dictyostelium* papers are identified from PubMed and their full text is retrieved where an open-access copy exists, with a curator able to supply a licensed copy otherwise. A language model then drafts the gene summaries, Gene Ontology terms, phenotypes and interactions that the paper reports, restricted to genes named in the text. The corresponding author receives a private link to a form holding those drafts, corrects or removes anything inaccurate, and submits. A curator then accepts or rejects each entry individually, or returns it with a question, and may continue the exchange until the annotation is resolved. Submitted entries appear on the gene record immediately, marked as provisional and awaiting curator review, and join the curated record once approved. Authors may sign in with ORCID so that the contribution is attributed to them. This approach follows community curation as practiced by PomBase through Canto [35, 36] and by WormBase through its author-curation pipeline [37], differing in that the author starts from a machine-drafted annotation rather than a blank form.

### Interoperability.

As the *Dictyostelium* MOD, dictyBase both consumes and contributes to community data resources. Its curated Gene Ontology annotations are exported in the standard GAF 2.2 format for contribution to the Gene Ontology Consortium, and its gene nomenclature and records are cross-referenced with NCBI and UniProt, maintaining consistent identifiers across these resources.

## Sustainability and future directions

### A low-cost model for community databases.

We designed dictyBase to retain broad coverage while lowering the cost of operation. Serving pre-computed static files from a single service removes the need for a database server and distributed microservices. The resulting system is less dependent on a single engineer, and both the software and data remain available for inspection and independent deployment. For communities without funding for a large engineering team, this architecture offers one way to maintain a specialized resource at modest cost. AI-assisted development further reduced the effort required in this case.

### Limitations.

This design also has limitations. It is optimized for a read-mostly resource at the query volumes typical of a specialized community. It is not intended, however, to support high-throughput transactional workloads or large, concurrent development teams. Features that depend on persistent user accounts or complex transactions are intentionally minimal, with reagent requests handled through a simple request workflow.

## Figures and Tables

**Figure 1:**
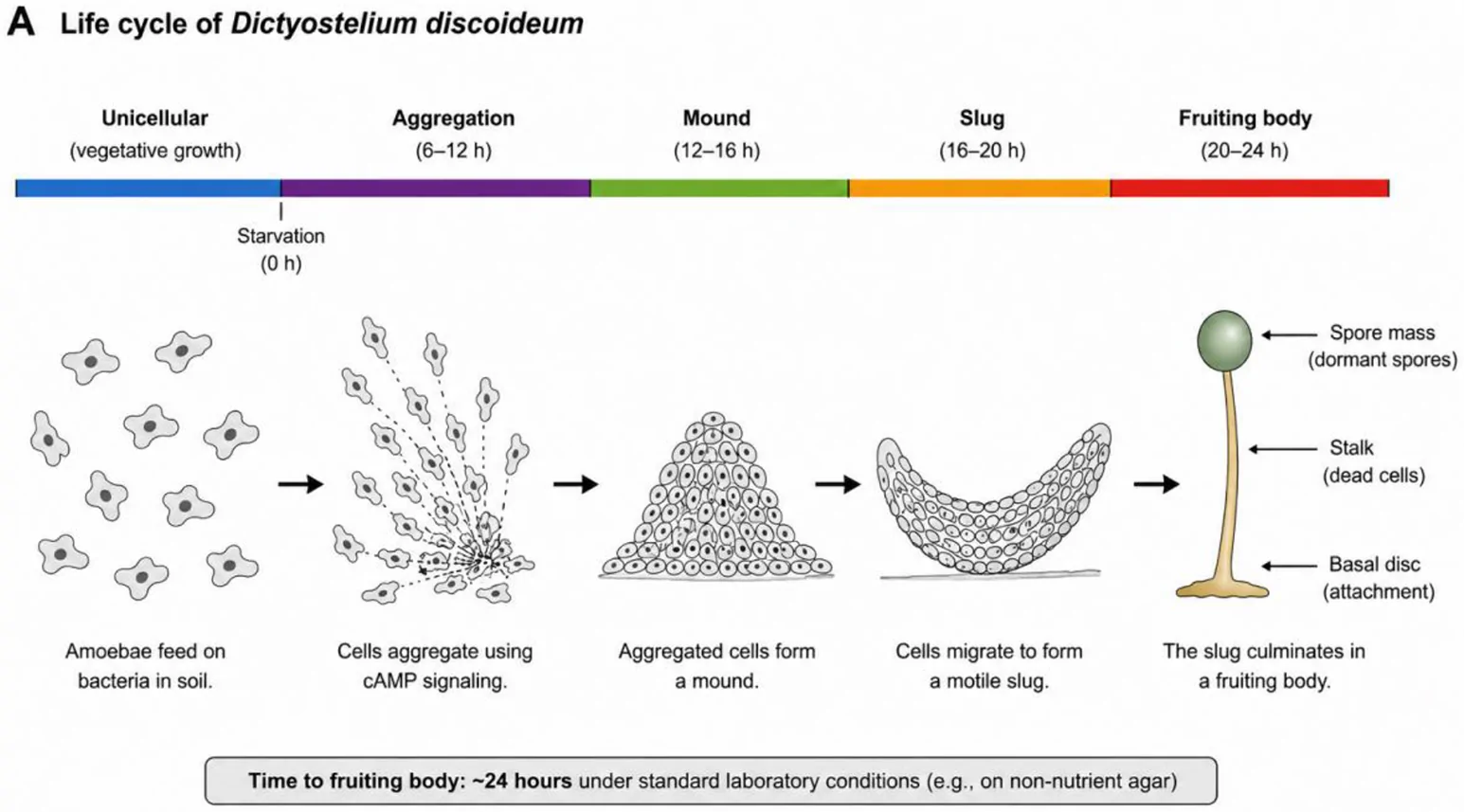
Schematic of the *Dictyostelium* life cycle. *Dictyostelium* feed on bacteria in the soil as unicellular amoebae, however if the food source becomes depleted, they begin a developmental cycle. Initially *Dictyostelium* undergo aggregation where they chemotax in response to a cAMP signal, ultimately forming the mound. Following the mound stage, *Dictyostelium* can form a motile slug that migrates to an optimal location via phototaxis and thermotaxis to culminate into a fruiting body. The fruiting body is composed of a basal disc and stalk made of dead cell, and a sorus that contains spores which can be distributed to germinate back into amoebae.

**Figure 2:**
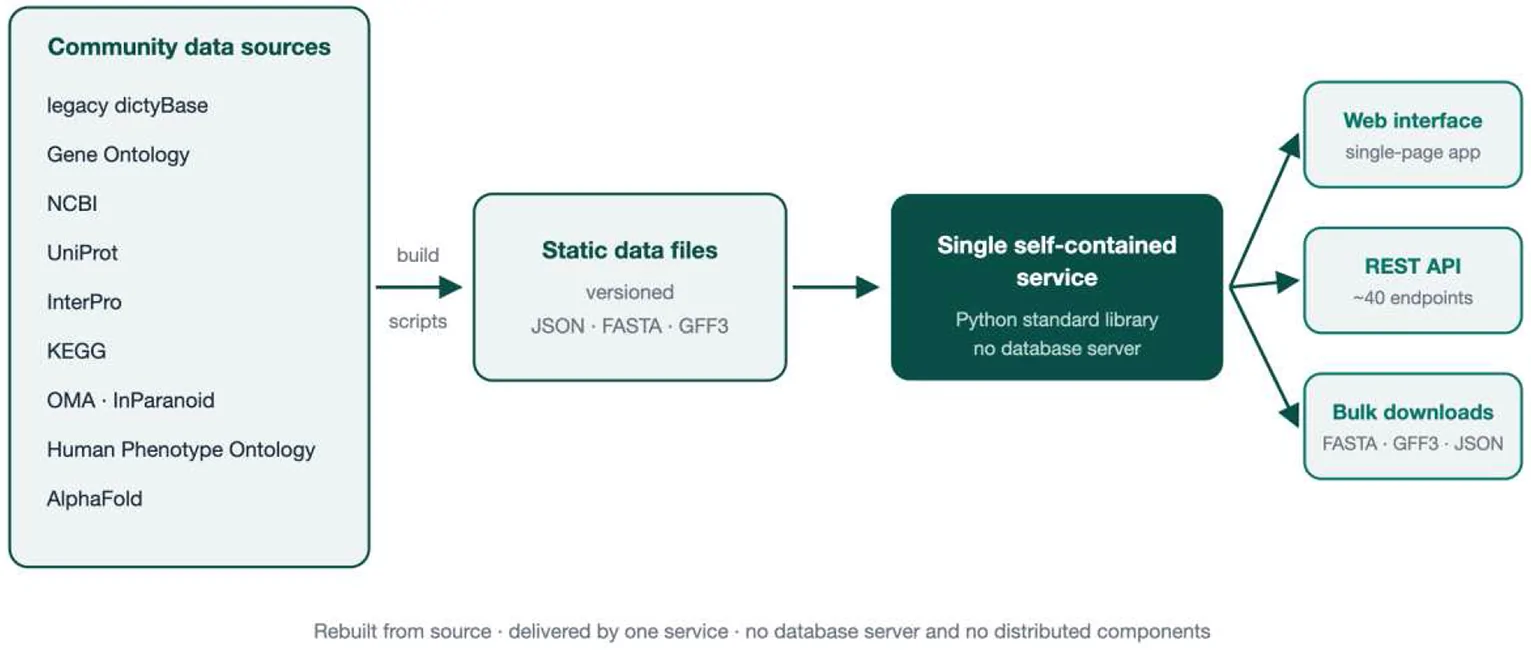
Architecture and data flow of the reimplemented dictyBase. Community data sources are rebuilt by a documented set of build scripts into versioned static data files in JSON, FASTA, and GFF3 format. A single self-contained service delivers those files to the web interface, a public REST API, and bulk downloads. No database server or distributed services are required.

**Figure 3.**
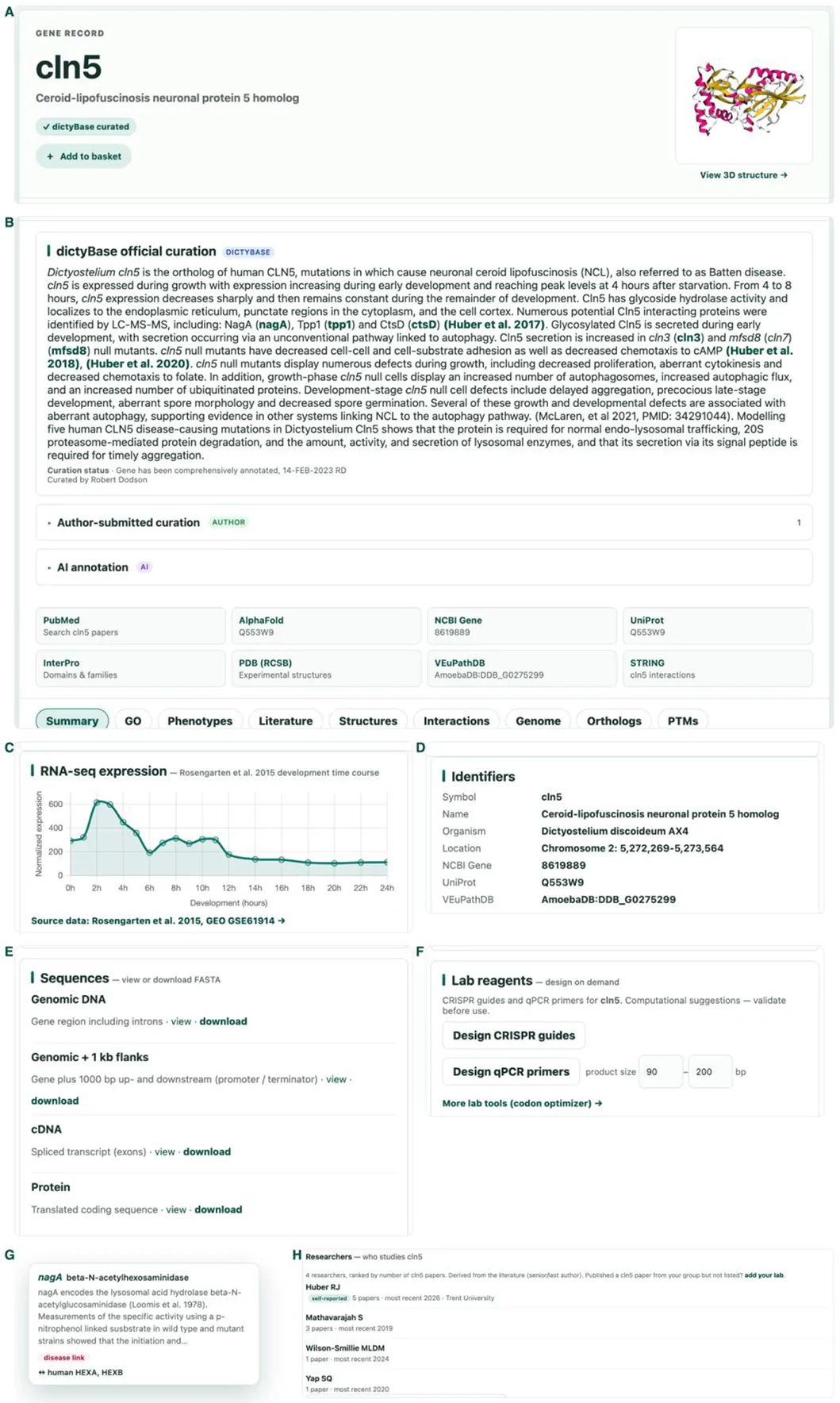
The dictyBase gene record, shown for *cln5*, the *Dictyostelium* homolog of human *CLN5*. **(A)** Gene identity: approved symbol, product name, an embedded AlphaFold predicted structure, and the control that adds a gene to the personal workspace. **(B)** Three separately badged annotation layers, expert dictyBase curation, author-submitted curation, and the machine-generated layer, above one-click cross-references to external resources and the tabbed record sections. **(C)** Developmental RNA-seq expression across the 24-hour time course. **(D)** Identifiers and cross-references. **(E)** Downloadable sequences in FASTA format. **(F)** Guide-RNA and quantitative-PCR primer design run directly from the record. **(G)** Hovering any gene symbol previews that gene without navigating away. **(H)** The Literature tab lists the investigators who have published on the gene, identified from the senior authors of its papers, with self-reported entries marked as such.

## Footnotes

Availability dictyBase is freely available at https://dicty.org with no registration required. Curated gene summaries are released under CC BY-NC 4.0; other data are redistributed under the terms of their source resources. The complete source code and data-build scripts are openly available at https://github.com/kmscaglione/dictyatduke (archived at Zenodo, https://doi.org/10.5281/zenodo.21812669) under the MIT License, allowing independent deployment and reuse.
